# Trade-off between motor performance and behavioural flexibility in the action selection of cricket escape behaviour

**DOI:** 10.1038/s41598-019-54555-7

**Published:** 2019-12-02

**Authors:** Nodoka Sato, Hisashi Shidara, Hiroto Ogawa

**Affiliations:** 1 Graduate School of Life Science, Hokkaido University, Sapporo, 060-0810 Japan; 2 Department of Biological Sciences, Faculty of Science, Hokkaido University, Sapporo, 060-0810 Japan

**Keywords:** Decision, Decision

## Abstract

To survive a predator’s attack successfully, animals choose appropriate actions from multiple escape responses. The motor performance of escape response governs successful survival, which implies that the action selection in escape behaviour is based on the trade-off between competing behavioural benefits. Thus, quantitative assessment of motor performance will shed light on the biological basis of decision-making. To explore the trade-off underlying the action selection, we focused on two distinct wind-elicited escape responses of crickets, running and jumping. We first hypothesized a trade-off between speed and directional accuracy. This hypothesis was rejected because crickets could control the escape direction in jumping as precisely as in running; further, jumping had advantages with regard to escape speed. Next, we assumed behavioural flexibility, including responsiveness to additional predator’s attacks, as a benefit of running. The double stimulus experiment revealed that crickets running in the first response could respond more frequently to a second stimulus and control the movement direction more precisely compared to when they chose jumping for the first response. These data suggest that not only the motor performance but also the future adaptability of subsequent behaviours are considered as behavioural benefits, which may be used for choosing appropriate escape reactions.

## Introduction

It is necessary to choose actions in every situation in daily life. Animals do not always enact the same reaction to a certain stimulus, in that they decide on the appropriate behaviour depending on the situation, external stimulus^1–5^, and internal state^6,7^. Numerous studies in behavioural economics have revealed that this decision-making is based on the trade-off between the amount of expected benefit and effort for the chosen behaviour^8–10^. Thus far, most studies on animals have focused on the relationship between the choice in a learned task and the expected value of rewards such as food intake resulting from their reactions^11–13^. In this context, the amount of benefit is related with neither the motor characteristics nor performance of the chosen behaviour. In contrast, the performance of innate behaviours in natural environments is directly linked to their benefits, such as survival rate against predators^14,15^ and mating success rate^16,17^. Therefore, quantitative assessment of the performance of selected behaviours is necessary to understand the innate mechanisms of decision-making in animals.

Escape actions that directly affect the survival of animals^18^ are a universal innate behaviour exhibited by various species, including vertebrates and invertebrates^19–21^. The benefits of escape behaviour can be regarded as the characteristics of its performance which make escape more successful. To successfully evade predator attacks, the speed of the escape movement is considered to be one of the most important features^15,22^. However, several animal species exhibit multiple types of escape actions; some actions are slower than others^22–29^. This suggests that slower escape reactions may have other benefits to compensate for speed. For instance, fish displays the following two different types of escape behaviour: C-turn behaviour triggered by firing of giant nerve cells identified as Mauthner cells (M-cells) and another without M-cell activity. The C-turn allows fish to escape at high speed^30,31^, but the stereotypic locomotion of the C-turn is considered a disadvantage because it makes the prey’s movement easily predictable by the predator^32^. Meanwhile, the slower response not mediated by M-cells is more variable in escape direction than that of the C-turn^26,27^, making it harder to predict by predators. Thus, the decision-making of fish for escape behaviour appears to be based on the trade-off between speed and unpredictability. However, little is known on the benefits of the trade-off underlying the decision-making in the context of innate behaviour. To address this question, a quantitative and comprehensive analysis of motor performance in innate behaviours is required.

Crickets exhibit several innate escape actions including running and jumping in response to a short air current detected as predator approach using the cercal sensory system^33–35^. Jumping responses in crickets are similar to those in locusts in which animals escape exclusively in a forward direction with high speed^36–39^. These features of jumping seem to be advantageous for a successful escape. However, crickets exhibit running responses more frequently than jumping for escape elicited by an air stimulus^40^, suggesting that running for escape may have advantages other than speed. Our previous experiments using a treadmill system revealed that crickets run away from the stimulus source in a direction opposite to that of the airflow^41^. Their direction of movement in the wind-elicited escape response is precisely controlled by, and dependent on, the stimulus angle. The accurate control of movement direction reported in various species^19,20,42^ is critical for escape behaviour to increase distance from the predator. Thus, a trade-off between speed and directional control possibly exists in the cricket escape behaviour: jumping would allow crickets to escape quicker but to control their movement direction less accurately than running.

Here, we hypothesized that running and jumping of the cricket would have distinct behavioural benefits such as speed and directional accuracy. To test this hypothesis, we quantitatively assessed several aspects of motor performance of running and jumping and examined distinct benefits of these escape actions. Furthermore, we focused on behavioural flexibility as another important benefit for successful escape. Even if animals successfully avoid the first attack, predators may continue to chase and attack them^43^. In this case, it is important for the prey animals to flexibly adapt their behaviour in response to additional attacks. The crickets running away may be able to change their movement in response to additional attacks but jumping ones cannot. To mimic the predator’s chase, we applied two successive air currents and examined the response to the second stimulus that was applied during/after the running or jumping elicited by the first stimulus. Our results proposed that the trade-off in motor performance of the reaction as well as flexibility for taking subsequent actions could be involved in decision-making in escape behaviour.

## Results

### Differences in speed and movement distance between running and jump

**Figure 1:**
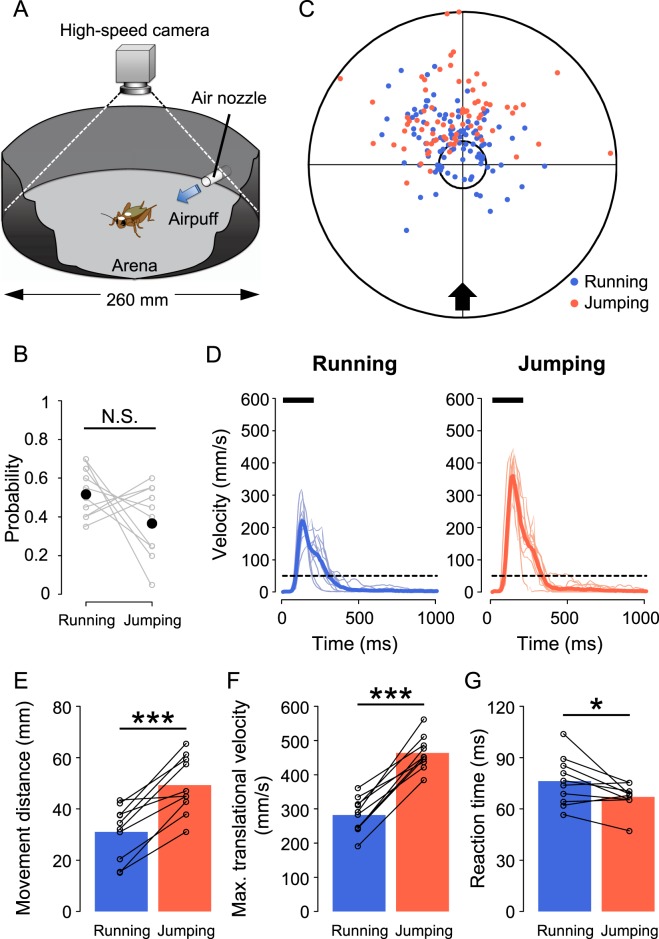
Difference in metric locomotor parameters between running and jumping. (**A**) Experimental apparatus for monitoring crickets’ movement. A cricket with two white markers on its dorsal surface was placed in the centre of the circular arena. Air current stimulus was applied horizontally to the cricket from one nozzle attached to the wall (blue arrow). The cricket’s movement was monitored with a high-speed digital camera at 120 fps from above. (**B**) Probabilities of running and jumping in response to the air-current stimulus. Gray open circles connected with lines represent the response probabilities for each individual, and black filled circles represent the mean of the probabilities for all individuals. N.S., not significant, Wilcoxon signed-rank test. N = 10 animals. (**C**) Finish points of running (blue) and jumping (red) for all trials. The black arrow indicates the direction of the air current. The small circle indicates the range of starting positions of the crickets enclosed within a beaker, and the large circle indicates the wall of the experimental arena. (**D**) Time course of translational velocity in running (left) and jumping (right). Thin coloured lines represent the time course of mean velocity for each individual, and thick coloured lines represent the average of the mean time courses for all individuals. Black bars indicate the stimulus duration. Dotted lines indicate the thresholds for criteria of the response. (**E**–**G**) Averages of mean movement distance (**E**), mean maximum translational velocity (**F**), and mean reaction time (**G**) for all individuals. Black open circles connected with lines indicate the mean value for each individual. *p < 0.05, **p < 0.01, ***p < 0.001, paired t-test. N = 10 animals.

We measured movement trajectory and several motor parameters from high-speed digital video images of the cricket escape behaviours (Fig. 1A). Crickets exhibited two distinct wind-elicited escape behaviours, either running or jumping, which were easily distinguished from each other by frame check of video images (Supplementary Fig. S1, See Methods). To compare motor performances such as the speed and reaction time between running and jumping within an individual cricket, we applied 20 airflow stimuli for each individual repeatedly (Supplementary Fig. S2A). The stimulus (618 mm/s) used in our previous study exclusively elicited the running response, but rarely caused jumping^40^. Then, in order to elicit not only running but also jumping for each cricket, a faster stimulus (834 mm/s) was used in this study. Running was observed with a probability of 53% (106/200 trials) and jumping was with a probability of 38% (76/200 trials; Fig. 1B). We rarely observed complex behaviours combined with running and jumping, such as jumping after running or turning. Neither response probability nor behavioural selection of “running or jumping” depended on the trial order, meaning that the crickets did not habituate to the repetitive stimulation throughout our experiments (Supplementary Fig. S2C,D). The fact that crickets chose running or jumping from time to time suggests that they would perform decision-making for behavioural selection.

Distribution of the arrival points of the escape responses indicated that jumping enabled crickets to escape farther away from the stimulus source than did running (Fig. 1C). In addition, the translational velocities were significantly higher in jumping than in running (Fig. 1D). To clarify these differences quantitatively, the metric locomotor parameters including movement distance, maximum translational velocity, and reaction time were compared between running and jumping (Fig. 1E,G, Supplementary Fig. S3A–C). Indeed, crickets moved longer distances with higher velocity on jumping than on running (Fig. 1E,F, Supplementary Fig. S3A,B). Furthermore, crickets initiated jumping significantly quicker than running (Fig. 1G, Supplementary Fig. S3C). These results demonstrated that compared to running, jumping had advantages in terms of speed and distance.

### Accuracies of the moving direction control in running and jump

**Figure 2:**
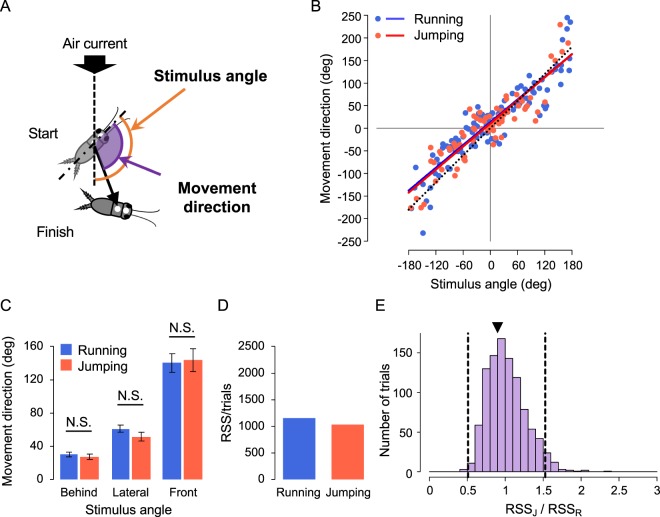
Movement direction for running and jumping. (**A**) Diagram showing the definition of movement direction (purple) and stimulus angle (orange). (**B**) Relationships between movement direction and stimulus angle in running (blue) and jumping (red). Lines represent linear regression lines for the data for running (blue) or jumping (red). The black dotted line indicates line of y = x. (**C**) Average absolute angular values of the movement direction in running and jumping. The data were divided into three ranges of stimulus angles that corresponded to behind, lateral, and front (see Materials and Methods). Error bars indicate ± SEM. N.S. not significant, Student t-test. (**D**) RSS/trials values of regression lines for movement direction. (**E**) Distribution of RSS_J_/RSS_R_ values for the shuffled data. The area between the two dashed lines indicates the range of the mean ± 2SD of the shuffled data, and the black triangle indicates the RSS_J_/RSS_R_ of the real (non-shuffled) data. The data obtained from all the trials in which crickets responded were used for these analyses.

Next, to investigate how accurately crickets controlled escape direction during running and jumping, movement direction against the stimulus angle was compared between these two reactions. Here, movement direction was defined as the angle between the body axis at the start point and a line connecting the start to finish points of the response. The stimulus angle was defined as the orientation of the cricket’s body axis at the start point against the stimulus source (Fig. 2A). In this experiment, the crickets were stimulated by airflow from any direction randomly, and both running and jumping were elicited by stimuli from all directions (Fig. 2B, Supplementary Fig. S4). This enabled us to determine whether the crickets controlled the direction of their movement responses to the stimulus from various directions in each response.

The regression line for the plot of movement direction against stimulus angle was close to y = x in running (blue in Fig. 2B), which was similar to those of previous results^40,41^, meaning that crickets moved precisely in the opposite direction to that of the stimulus source. Unexpectedly, this linear relationship was also observed in jumping behaviour (red in Fig. 2B). Both slopes of the regression lines for running and jumping were almost 1 (0.839 for running and 0.851 for jumping, Fig. 2B), and the correlation coefficients (R^2^) of these linear relationships had similar values (0.840 for running and 0.820 for jumping). Further, we divided all trials into three categories, “behind”, “lateral”, and “front”, based on the stimulus angle and compared the magnitude of movement direction (Fig. 2C). There was no difference in the angular magnitude of the movement direction between running and jumping for all categories. Thus, the crickets controlled not only running but also jumping to escape in the opposite direction to the stimulation source.

To statistically test the variability of movement direction against the stimulus angle, the mean of the residual sum of square (RSS/trials) for all plots from the regression line was calculated for each response (1152 for running and 1029 for jumping, Fig. 2D). To assess the difference in the RSS/trials between running and jumping, we performed the permutation test for the ratio of the RSS/trials of the responses (RSS_J_/RSS_R_), which was used as the index indicating the variability in directional control. Figure 2E shows the distribution of RSS_J_/RSS_R_ in all shuffled datasets. The RSS_J_/RSS_R_ value of the original data, indicated by a black triangle in Fig. 2E, was 0.893 which was close to 1 and within the range of the mean ± 2S D of the shuffled data. Thus, the variability of movement directions in running and jumping were not significantly different. Taken together, these data imply that crickets controlled the movement direction in jumping as accurately as in running.

### Dependency of the turn angle on stimulus angle in running and jump

**Figure 3:**
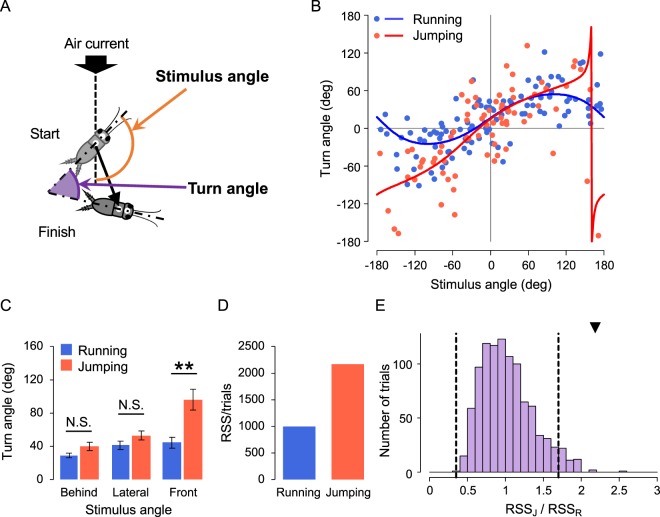
Movement turn angle for running and jumping. (**A**) Diagram showing the definition of the turn angle (purple) and stimulus angle (orange). (**B**) Relationships between the turn angle and stimulus angle in running (blue) and jumping (red). Curves represent circular-circular regression curves. (**C**) Average absolute angular values of the turn angle in running and jumping. The data were divided into three ranges of stimulus angles corresponding to behind, lateral, and front. Error bars indicate ± SEM. **p < 0.01, N.S. not significant, Student t-test. (**D**) RSS/trials values of regression curves for the turn angle. (**E**) Distribution of RSS_J_/RSS_R_ values for the shuffled data. The area between two dashed lines indicates the range of the mean ± 2SD of the shuffled data, and the black triangle indicates the RSS_J_/RSS_R_ of the real data. The data obtained from all the trials in which crickets responded were used for these analyses.

We also investigated the turn angle, which was defined as the orientation where the crickets turned (Fig. 3A), as the turn angle was also regulated depending on the stimulus angle, similar to movement direction^40,41^. In contrast to movement direction, the plots of turn angle for jumping (red in Fig. 3B) were distributed more widely than those for running (blue in Fig. 3B). In particular, the turn angles for the range of the stimulus angle from the front (around ± 180°) were different in their distribution between running and jumping. Unlike the responses to stimuli from the lateral direction and behind, the magnitude of the turn angle for the stimulus from the front was significantly larger in jumping than in running (Fig. 3C). This suggested that stimuli from the front caused larger turning in jumping than in running.

To examine the relationship between the turn angle and stimulus angle like the movement direction, we performed circular regression analyses. The regression curve of running (blue in Fig. 3B) differed from that of jumping (red in Fig. 3B), and the R^2^ value for the curve of jumping was smaller than that for running (0.858 for running and 0.745 for jumping). These results implied differences in the dependency of the turn angle on the stimulus between running and jumping. To test the variability of the turn angle against the stimulus angle, the RSS/trials for the plots from the regression curves were calculated. The mean RSS/trials for jumping was approximately twice as large as that for running (996 for running and 2165 for jumping, Fig. 3D). When we performed the permutation test and analysis of movement direction, the RSS_J_/RSS_R_ of the original data was much larger than the mean ± 2SD of the shuffled data (Fig. 3E), indicating that the turn angle in jumping was more varied than in running. These results demonstrated that the turning movement in jumping was not as tightly controlled with regard to the stimulus angle compared to that in running.

We assumed that the action selection of either running or jumping in the escape response would be based on the trade-off between speed and directional accuracy, but we did not observe such a trade-off. Jumping had advantages over running in terms of movement distance, maximum translational velocity, and reaction time, all of which reflected the escape speed. In contrast, the movement direction was equally precisely controlled in both running and jumping. Taken together, crickets could accurately move faster, further, and more quickly in the opposite direction to the stimulus in jumping. Thus, jumping was considered a more appropriate response than running for successful escape. Yet, why do crickets choose running as an escape response? We observed another difference in the variability of the turn angle, which was not directly related to the success rate of escape.

### Benefits of running in response to double stimulation with short intervals

We assumed flexibility as a benefit of running compared to jumping when crickets received continuous stimuli. Using three types of stimulation, we compared running and jumping in terms of behavioural flexibility (Supplementary Fig. S2B). For double stimulation, two air puffs with shorter duration (velocity: 834 m/s; duration: 100 ms) than that used in the single stimulus experiment were successively delivered from the same nozzle with short intervals. These shorter stimuli also elicited running and jumping equally as the first response (Supplementary Fig. S5A), and the difference in motor performance between running and jumping in “Single” was consistent with those in the single stimulus experiment (Supplementary Fig. S6), although no significant difference was observed in reaction time between running and jumping (Supplementary Fig. S6C). Since the crickets moved in precisely the opposite direction to the air nozzle regardless of running and jumping (Fig. 2B), crickets that are still located on the straight course of airflow even after responding to the first stimulus were also stimulated by the second stimulus (Supplementary Fig. S7A).

**Figure 4:**
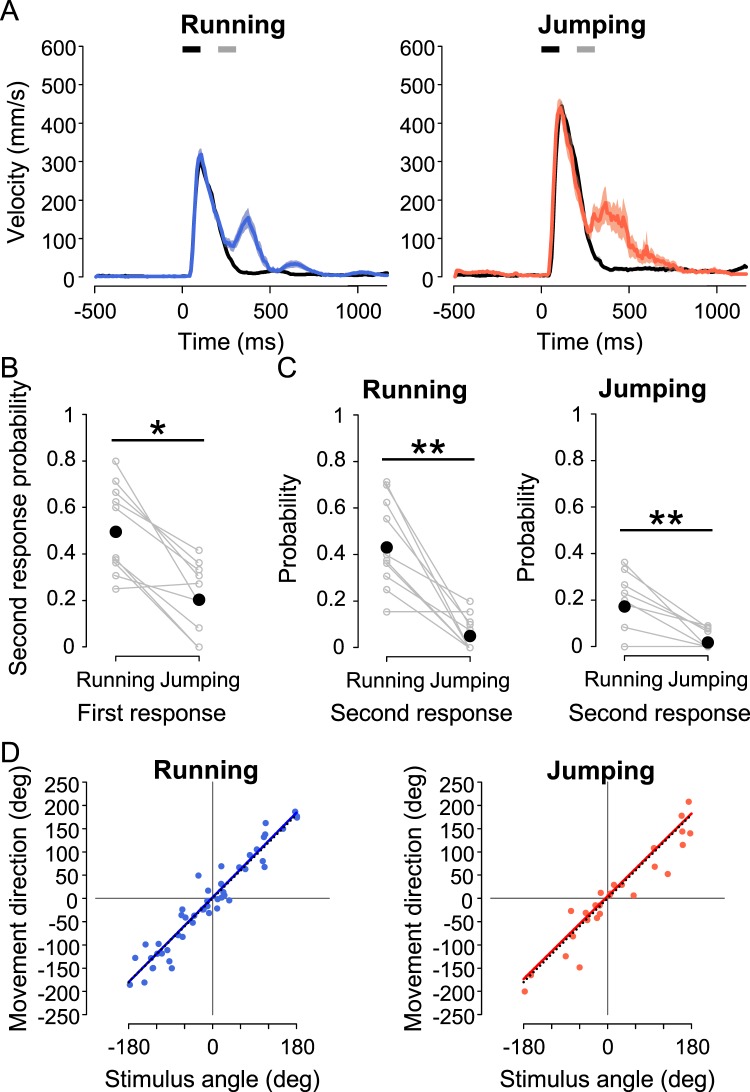
Response to successive double stimulation of short intervals. (**A**) Time course of translational velocity in the escape responses in the “Single” and “Double 100” experiments. Black lines indicate the average of the mean time course for all trials of running (left) and jumping (right) in “Single”; coloured lines indicate those for trials with responses to both the first and second stimuli in “Double 100”, which are divided into running (left) or jumping (right) as the first response. Shaded areas represent range of mean ± SEM. Black and gray bars indicate the first and second stimuli, respectively. (**B**,**C**) Probabilities of the second response. Probability of second response including running and jumping during each behaviour as the first response (**B**), and those of running and jumping as the second response following the first response of running (left) or jumping (right) (**C**). Gray open circles connected with lines represent the response probabilities for each individual; black filled circles represent the mean of the probability in all individuals. *p < 0.05, **p < 0.01, Wilcoxon signed-rank test. N = 10 animals. (**D**) Relationships between the movement direction of whole escape behaviour and the first stimulus angle in the trials responding to both stimuli in “Double 100”, which is divided into running (left) and jumping (right) as the first response. Coloured lines represent linear regression lines for the data of running (blue) or jumping (red), and black dotted lines indicate lines of y = x.

First, we examined the responsiveness to the second stimulus applied at a 100-ms interval (“Double 100”). In this stimulation, the second air-puff was applied before the end of the initial responses; the duration of the initial responses was >250 ms in running and >300 ms in jumping after the first stimulus onset, respectively (Supplementary Figs. S3C,D, S6C,D). Figure 4A shows the typical time course of the translational velocity if the crickets responded to the second stimulus during running (left) or jumping (right). Although the probabilities of running and jumping to the first stimulus were almost equal (centre in Supplementary Fig. S5A), those for the second stimulus were affected by the choice of the first response (Fig. 4B,C). The crickets that ran in response to the first stimulus responded to the second stimulus with higher probability than those that jumped (Fig. 4B). Regardless of the first response choice, the probability of jumping as the second response was lower than that of running (Fig. 4C). Thus, crickets responded to the second stimulus more frequently during running than jumping and were likely to choose running in response to the second stimulus.

**Figure 5:**
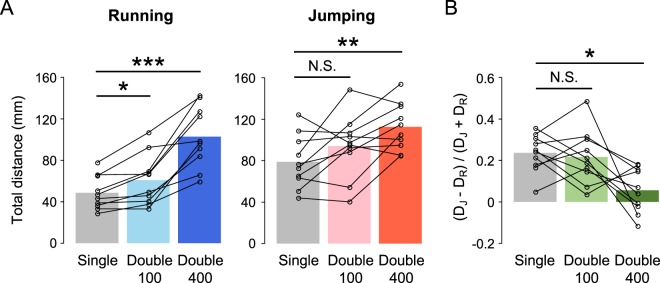
Total movement distance in escape responses to double stimulation. (**A**) The total movement distances of entire escape locomotion in trials where running (left) or jumping (right) was elicited by the first stimulus. Bars indicate the average of the mean value for all individuals in “Single” (gray), “Double 100” (light coloured), and “Double 400” (dark coloured) stimulation experiments. Open circles connected with lines represent the mean values for each individual. *p < 0.05, **p < 0.01, ***p < 0.001, N.S. not significant, paired t-test with Bonferroni correction for multiple comparisons. (**B**) The normalized differences in total movement distance between running and jumping responses to the first stimulus. Bars indicate the average of the mean value for all individuals in “Single” (gray), “Double 100” (light green), and “Double 400” (dark green) stimulation experiments. *p < 0.05, N.S. not significant, Wilcoxon signed-rank test with Bonferroni correction for multiple comparisons. N = 10 animals.

To examine the effects of the first choice on escape behaviour in “Double 100” experiments, we compared the difference in total movement distance between running and jumping as the first response. In both running and jumping trials in response to the first stimulus, the distribution of the finish points in “Double 100” was similar to that in “Single” (Supplementary Fig. S5B). However, the total movement distance of the crickets running as the first response in “Double 100” was significantly longer than that in “Single” (left in Fig. 5A). In the case of jumping response to the first stimulus, there was no difference in the total movement distance between “Single” and “Double 100” (right in Fig. 5A). In addition, we assessed whether the movement direction of whole escape behaviour was affected by responding to the second stimulus. When crickets responded to the second stimulus, the movement direction measured at the finish points was controlled relative to the angle of the first stimulus in both running and jumping responses, as precisely as that in “Single”. In “Double 100”, the slope and R^2^ value of the regression lines were 1.012 and 0.927 for running, and 0.963 and 0.903 for jumping, respectively; in “Single”, they were 0.990 and 0.896 for running, and 0.987 and 0.881 for jumping, respectively (Fig. 4D, Supplementary Fig. S6E), implying little advantage of running in directional control.

### Benefits of running in response to double stimulation with long intervals

**Figure 6:**
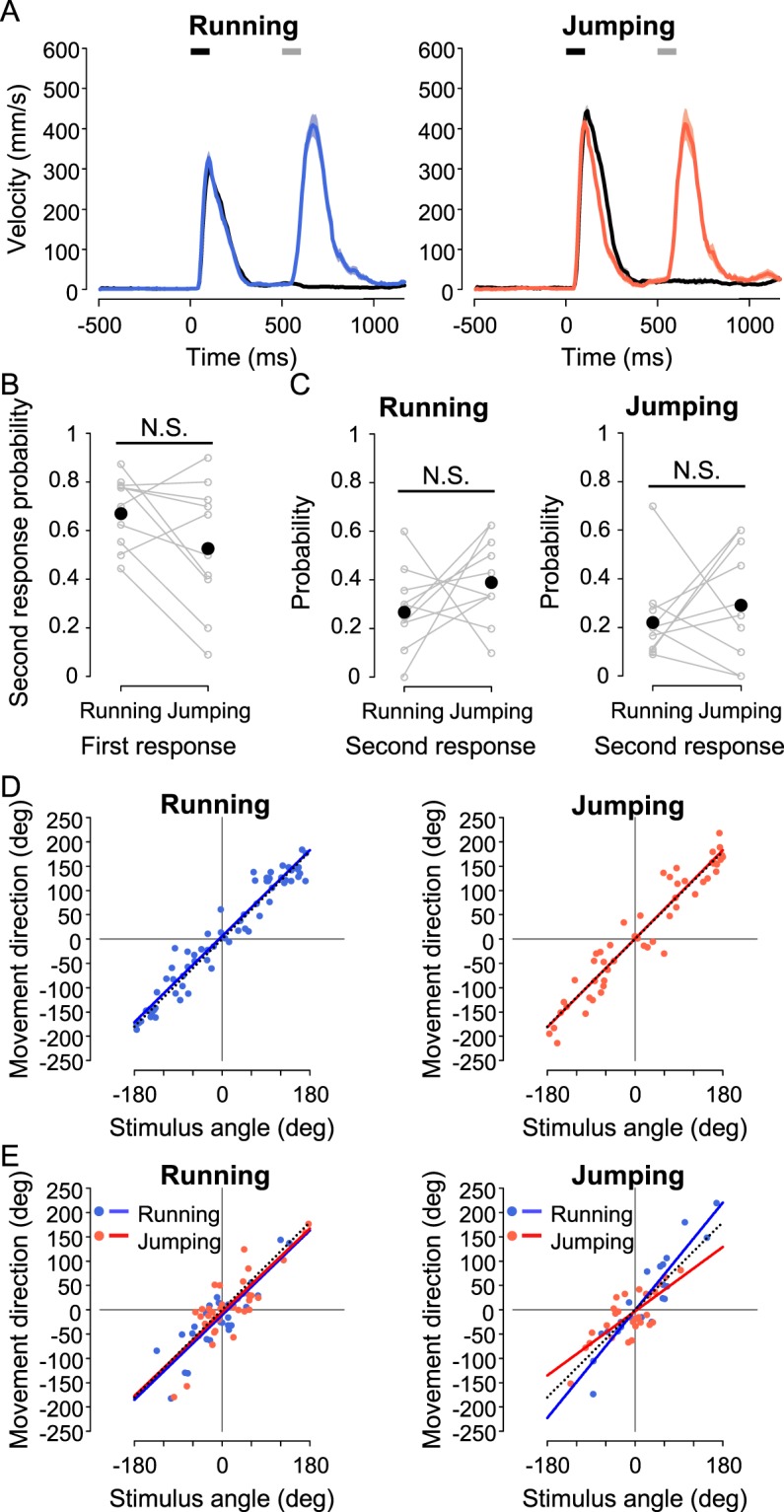
Response to successive double stimulation of long intervals. (**A**) Time course of translational velocity in escape responses in the “Single” and “Double 400” experiments. Black lines indicate the average of the mean time course for all trials of running (left) and jumping (right) in “Single”; coloured lines indicate those for trials with responses to both the first and second stimuli in “Double 400”, which are divided into running (left) or jumping (right) as the first response. Shaded areas represent range of mean ± SEM. Black and gray bars indicate the first and second stimuli, respectively. (**B**,**C**) Probabilities of the second response. Probability of second response including running and jumping after each behaviour as the first response (**B**), and those of running and jumping as a second response after a first response of running (left) or jumping (right) (**C**). Gray open circles connected with lines represent the response probabilities for each individual, and black filled circles represent the mean of the probability for all individuals. N.S., not significant, Wilcoxon signed-rank test. N = 10 animals. (**D**,**E**) Relationships between the movement direction and stimulus angle of the first response (**D**) and second response (**E**) in the trials with responses to both stimuli in “Double 400”, which are divided into running (left) and jumping (right) as the first response. Coloured lines represent linear regression lines for the data for running (blue) or jumping (red), and black dotted lines indicate lines of y = x.

The high responsiveness to successive stimuli was considered as the benefit of running. It was possible that this benefit would be specific to “during locomotion”. To test this possibility, we investigated the response to the second stimulus applied with a 400-ms interval (“Double 400”). In this experiment, the second air-puff was given just after the initial response finished (Supplementary Figs. S3C,D, S6C,D). Indeed, when the crickets responded to the second stimulus, a distinctive response was elicited both after running and jumping (Fig. 6A). There was no difference in responsiveness to the second stimulus between running and jumping trials for the initial response (Fig. 6B). In addition, both running and jumping were chosen equally as the second response independent of the initial reactions, similar to the first choice (Fig. 6C, Supplementary Fig. S5A). Furthermore, the distance from the air-current course at the second stimulated location did not affect the selection of the second reaction (Supplementary Fig. S7B). Thus, the benefit of responsiveness to successive stimuli in running was specific to cases when crickets were successively stimulated during locomotion.

We also examined the total movement distance for the whole escape behaviour in “Double 400” experiments for each individual. The distribution of the finish points indicated that “Double 400” crickets moved farther from the start position than those in “Single” for both running and jumping trials (Supplementary Fig. S5C). Indeed, the total movement distance in “Double 400” was significantly longer than that of “Single”, independent of the initial response choice (Fig. 5A). The difference in the total distance of running (D_R_) and jumping (D_J_) significantly decreased in “Double 400” compared to that in “Single”. In contrast, that difference between D_R_ and D_J_ in “Double 100” was not significantly different from that in “Single” (Fig. 5B). Thus, the total travel distance increased in “Double 400” experiments regardless of which type of behaviour crickets chose for the first response, but the difference in the distance between running and jumping was reduced by the response to additional stimulus.

Finally, we investigated directional control when crickets responded to the second stimulus in “Double 400”. The position of the crickets when they received the second stimulus has little effect on the performance of the second response (Supplementary Fig. S7C–E). We analysed the relationship between the movement direction measured at the finish points of the first or second response and the angles of the first or second stimulus (Fig. 6D,E). In terms of the first response, the movement direction was precisely controlled against the first stimulus angle regardless of behaviour. The slopes and R^2^ values of the linear regression lines were 0.965 and 0.932 for running, and 0.988 and 0.887 for jumping, respectively (Fig. 6D). These results were similar to those shown in Fig. 3B. However, the plots of the movement direction of the second response showed different distributions between running and jumping trials for the first response (Fig. 6E). When the crickets chose running as the first response, the movement direction in both the second running and second jumping responses was controlled against the second stimulus angle with large variability. The slopes and R^2^ of the regression lines were 0.9683 and 0.7524 for running, and 0.9565 and 0.6749 for jumping, respectively (left in Fig. 6E). In contrast, if the cricket jumped in response to the first stimulus, the directional control of running following jumping was different to that of jumping following jumping. The slopes and R^2^ values of the linear regression lines were 1.230 and 0.848 for running, and 0.735 and 0.574 for jumping, respectively (right in Fig. 6E). Thus, choosing jumping as the initial response affected directional control during the subsequent behaviour.

Collectively, if the second stimulus was applied after termination of the initial response, the crickets could respond to it regardless of initial response behaviours, but the disadvantage in movement distance of running was lost. Furthermore, the choice to jump in response to the first stimulus was inferior to that to running in terms of directional control in the second response.

## Discussion

The escape response is an innate defensive behaviour to survive in the face of predators^3,19,20^ and is predominantly chosen over other behaviours in emergent situations^44–46^. The ultimate goal of escape behaviour is successful escape from predators, and the motor performance of escape has critical impact on the survival rate. Here, we quantitatively assessed the motor performance of two distinct escape responses of crickets, running and jumping, to clarify the behavioural benefits of each response that would be involved in the action selection in the escape behaviour. Firstly, we hypothesized that running had advantages in the control of movement direction in exchange for the moving speed. However, crickets controlled their movement direction in jumping as precisely as in running, which rejected the possibility of the trade-off between the speed and accuracy of directionality. Next, we considered behavioural flexibility as another advantage of running. Double stimulus experiment revealed that running had the advantages of responsiveness and precise control of movement direction in the case of continuous stimulation and that the disadvantage of shorter travel distance in running as the first response was compensated by the following response. Thus, crickets that chose running in response to the first attack were able to respond flexibly to further attacks, which may be a benefit of running as an escape behaviour. The action selection in the wind-elicited escape behaviour of the cricket may involve a trade-off between the farther and faster escape in the first response and the adjustability of subsequent responses to persistent attacks by a predator.

In animals’ locomotive behaviour, moving speed often conflicts with the precise control of movement direction^27^. For example, the fact that the locust can jump only forward^39^ also suggests an incompatibility between the moving speed and directional control. In the crickets’ wind-elicited escape responses, however, they were able to control the movement direction of jump that allows crickets faster away. Surprisingly, the crickets jumped sideways and backwards. The crickets’ jumping system may be physically different from that of locusts^47^. In the rapid escape responses of flies and cockroaches, movement direction is precisely controlled^42,48^, with which our results were consistent. Variability in movement direction is considered another motor benefit because it would provide unpredictability to the attacking predator^27,43^. However, there was no difference in directional variability between running and jumping (Fig. 2E). Taken together, comparison in the movement direction failed to explain the benefits of running that would trade-off for the advantages of speed and movement distance in jumping.

Thus, an alternative explanation beyond simple motor performance in escape strategies was proposed; namely, behavioural flexibility. Responsiveness to subsequent predator attacks during the escape response to the first attack is critical for successful escape. The crickets running in response to the first stimulus more frequently responded to the second stimulus than jumping ones (Fig. 4B), indicating that the behavioural flexibility would be considered as an advantage of running which allows crickets to respond to additional attack. Since a running cricket has any one of its legs on the ground, it is able to change its movement if receiving the second stimulus. In contrast, it would be difficult for the crickets to respond to the stimulus during jumping because they were away from the ground. Once the escape response was terminated, the behavioural choice for the initial response did not affect that of the second response (Fig. 6B), which also supported the conclusion that the responsiveness especially during the movement is the advantage of running. Although research has revealed that a pre-stimulus affects behavioural performance in response to a subsequent alert stimulus^49^, there have been no reports focusing on behavioural flexibility following the first response. Our present results demonstrated that animals’ action selection affects subsequent behaviour.

In this study, we focused on observable motor performance such as speed, direction, and response probability. Nevertheless, other factors that are difficult to measure such as energy cost may affect decision-making. In crickets, the energy cost to run is considered smaller than that to jump^34,36^, thus running may have more cost advantage than that of jumping. For jumping to take off the ground, the heavier crickets would need larger energy, so would more frequently choose running escape rather than jumping. However, the selection ratio of running and jumping did not depend on the animals’ weights (Supplementary Fig. S2E,F). In addition, the crickets that are repeatedly stimulated would lose more energy, so may also choose running rather than jumping as in later trials. But, neither the response probability nor the ratio of running to jumping was changed throughout the experiment (Supplementary Fig. S2C,D). These results suggest that energy cost may not be critical for decision-making of escape behaviour. For action selection in emergency situations of life or death^10,50,51^, lower energy cost would be an insignificant benefit and the motor performance including speed, directional control, and flexibility are considered to be more important factors.

Our results revealed that responsiveness or flexibility following the initial reaction could be considered as behavioural benefits, which may be traded for the motor performance in innate behaviours. The beneficial value of behavioural performance changes according to the situation. For instance, the more rapidly a visual stimulus looms, the more likely *Drosophila* chooses escape behaviour with shorter reaction time in exchange for unstable flight^22,25,52^, suggesting that the value of escaping speed increases in more emergent situations. Similarly, crickets’ escape behaviours could be situation-dependent. These escape responses are crucial for survival; therefore, various parameters of sensory stimuli relate directly to behavioural outputs^25,27,38,46,53^. For wind-elicited escape in crickets, the effects of stimulus parameters such as intensity and direction on which behavioural outputs are dependent^33,41,54,55^ and the neural systems that process airflow information are well established^56,57^. Thus, this behaviour is useful for investigating sensory impacts on decision-making of action selection. Our results suggest the existence of trade-off between the motor performance of *in-situ* behaviour and the flexibility of subsequent behaviour and will contribute to understanding the neural substrates underlying decision-making in innate behaviours.

## Methods

### Experimental animals

Laboratory-bred adult male crickets (*Gryllus bimaculatus*) within two weeks after the imaginal moulting were used throughout experiments. They were reared under 12:12-hour light/dark conditions at a constant temperature of 27 °C and could freely access food and water. The guidelines of the Institutional Animal Care and Use Committee of the National University Corporation, Hokkaido University, Japan, specify no requirements for the treatment of insects in experiments. All experiments were conducted during the dark phase at room temperature (26–28 °C).

### Behavioural experiments

Cricket movement was monitored using a high-speed digital camera (CH130EX, Shodensha, Osaka, Japan) above the experimental arena (ø = 260 mm) throughout experiments (Fig. 1A). After anesthetized within a glass beaker cooled with ice (0 °C) for 10 min, crickets were marked with two white spots (ø = 3 mm) on the dorsal surface of the head and thorax in order to automatically detect the cricket’s movement on video images. After more than 30 minutes for recovery from the anesthetisation^58^, all experiments were performed. Crickets were placed in the centre of an arena within an inverted beaker (ø = 50 mm) covered with aluminium foil. After the beaker was carefully lifted, an air-current stimulus was immediately applied to the stationary cricket, and the wind-elicited response was recorded by the camera. Based on the video data (shutter speed, 1 ms; sampling rate, 120 frames/s; total recording duration, 1660 ms), the markers on the animal were automatically traced, and several locomotor parameters were measured with motion analysing software (Move-tr/2D, Library, Tokyo, Japan). To monitor the entire movement trajectory, we adopted 285.7 × 285.7 mm frame size in 1024 × 1024 pixel resolution, which covered the whole arena.

### Stimulation

The air-current stimulus was a short puff of nitrogen gas from a plastic nozzle (ø = 15 mm) connected to a pneumatic picopump (PV820, World Precision Instruments, Sarasota, FL, USA). One air-current nozzle was installed on the inside wall of the arena on the same horizontal plane as the animal (Fig. 1A). Since the crickets were oriented randomly within a beaker, the stimulus angles against the cricket were varied across trials. The air-current velocity at the centre of the arena was 834 mm/s throughout experiments. The travel time of the air to the centre was 14.6 ± 0.0798 ms, which was measured as a delay in stimulus-evoked ascending spikes of projection neurons that were extracellularly recorded (n = 2 animals, 10 trials) at the centre of the arena.

Two experiments were performed with different stimulations: single- and double stimulation (Supplementary Fig. S2A,B). In single stimulus experiment, only one air-current of 200-ms duration was applied in a trial. Twenty trials were repeatedly performed for each individual at inter-trial intervals of 60–90 sec (Supplementary Fig. S2A,B). In double stimulus experiment, we performed three sessions for each individual. To apply two successive stimuli during the initial response, air currents of 100-ms duration were used throughout all sessions. For the control (“Single”) session, only one stimulus was applied in a trial. In “Double 100” session, two successive puffs were applied at 100-ms intervals in a trial to examine the responsiveness to the stimulus during escape. In “Double 400” session, two successive puffs were applied at 400-ms intervals in a trial to examine the responsiveness to the stimulus just after escape. Twenty trials were performed for each session, so in total 60 trials were performed for each individual in the double stimulus experiments. Inter-trial intervals in each session were 60–90 sec. Between different sessions, crickets rested within a plastic container (138 mm × 220 mm × 135 mm) for about 10 min and freely accessed food and water. For each experiment, 10 animals were tested.

### Behavioural analysis

The wind-elicited response was analysed as reported previously^40^. Responding or not was defined based on translational velocity. If the translational velocity exceeded 10 mm/s during the period from the stimulus onset to 50 ms after the stimulus termination and the maximum translational velocity was greater than 50 mm/s, the cricket was considered to respond to the air current. If the cricket did not begin to move within the response definition period, which was 250 ms for single stimulus experiment or 150 ms for double stimulus experiment, that trial was considered as “no response”. In the double stimulus experiment of “Double 100”, because the second stimulus was applied to the cricket during movement elicited by the first stimulus, the cricket was considered to respond if the translational velocity increased during the 150-ms period after the second stimulus onset.

Wind-elicited responses were categorized as “jumping” or “running” according to leg movement during locomotion, which was confirmed visually for all response trials. If all six legs were off the ground simultaneously, that response was defined as “jumping”. If any one of six legs touched the ground during movement, that response was defined as “running” (Supplementary Fig. S1B). The response probability in the single stimulus experiment and the first response probability in the double stimulus experiment were calculated for each individual from the number of responses categorized as running or jumping over 20 trials (Fig. 1B, Supplementary Table S1, Supplementary Fig. S5A). Second response probability was calculated as the proportion of trials with response to the second stimulus relative to all trials with response to the first stimulus categorized as running or jumping (Figs. 4B,C and 6B,C).

Cricket movement during initial response was analysed as in previous studies^40,41,54^, of which the response start was defined as the time when the translational velocity exceeded 10 mm/s after stimulus onset; the finish was defined as the time when the velocity was less than 10 mm/s. Movement distance, maximum translational velocity, and reaction time were measured as metric locomotor parameters (Fig. 1E–G). The movement distance was measured as the entire trajectory of crickets. The reaction time was calculated by subtracting the mean travel time of the air current (mentioned above) from the response delay to operation of the picopump. Angular parameters including movement direction and turn angle were calculated based on the cricket’s body-axis, which is a vector connecting the thoracic and head markers. Movement direction was measured as the angle between the body axis at the start point of the response and a line connecting the thoracic markers at start and finish points (Fig. 2A). The turn angle was measured as the angle between the body axes at the start and finish points (Fig. 3A).

In the double stimulus experiment, the total movement distance of the cricket was measured from the onset of the first stimulus to the end of the video data (Fig. 5). In “Double 400”, the second response began after the first response had finished, which enabled measurement of stimulus angle and movement direction of the second response (Fig. 6E).

### Statistical analysis

R programming software (ver. 3.3.2, R Development Core Team) was used for all statistical analyses. Response probabilities were compared using Wilcoxon signed-rank test. Student t-test was used to compare running and jumping in metric locomotor parameters. For angular parameters, the absolute values (0°−180°) were compared between running and jumping in three different ranges of the stimulus angle, which corresponded to behind (−60° to 60°), lateral (60° to 120° and −60° to 120°), and front (120° to 180° and −120° to −180°) using Student t-test.

Since a strong linear correlation between stimulus angle and movement direction was observed as in previous studies^40,41,54,55^, movement direction was considered as non-circular data and linear regression analysis used as follows:

$$(Movement\,direction)=a(Stimulus\,angle)+b$$

Significance of the slope of the regression line (*α*) was tested. In contrast, to analyze the relationship between stimulus angle and turn angle that was considered circular data, circular-circular regression analysis was used as follows:

$$(Turn\,angle)=ta{n}^{-1}(\frac{A+Bcos(Stimulus\,angle)+Csin(Stimulus\,angle)}{a+bcos(Stimulus\,angle)+csin(Stimulus\,angle)})$$

Permutation test was adopted to assess the difference in variability between running and jumping in the distribution of movement direction or turn angle against stimulus angle. Initially, the mean value of residual sum of square (RSS/trials) was measured for each plot from the regression line or curve in running (RSS_R_) and jumping (RSS_J_), and the ratio of RSS_J_ to RSS_R_ was calculated. If the RSS_J_/RSS_R_ was larger than 1, it meant that the movement directions in jumping were more varied than those in running. Then, the category of the data, “running” and “jumping”, was shuffled 1,000 times, and the distribution of RSS_J_/RSS_R_ for all shuffled data was plotted. If RSS_J_/RSS_R_ for the original (non-shuffled) data was outside the range of the mean ± 2SD of the ratio for shuffled data, the difference in variability of the angular parameters between running and jumping was considered significant.

In the double stimulus experiment, to compare “Single” with “Double 100” or “Double 400” in total movement distance of individuals, paired t-test with Bonferroni correction for multiple comparisons was used. To determine whether the difference between running and jumping in total movement distance was altered by two stimulations, the ratio of the difference between the distance of running (D_R_) and jumping (D_J_) to their total sum was compared using Wilcoxon signed-rank test with Bonferroni correction for multiple comparisons.

## Supplementary information

Supplementary information

## Data Availability

The data that support the findings of this study are available from the corresponding author, H.O., upon reasonable request.
