# Supplementary material for: Trade-off between motor performance and behavioural flexibility in the action selection of cricket escape behaviour
Source: Sci Rep. 2019 Dec 2;9:18112. doi: 10.1038/s41598-019-54555-7 (PMC6889515; doi:10.1038/s41598-019-54555-7)
Supplement: Supplementary file 1 — Supplementary information [file 41598_2019_54555_MOESM1_ESM.pdf]

Trade-off between motor performance and behavioural flexibility in the action selection of cricket escape behaviour

Nodoka Sato, Hisashi Shidara & Hiroto Ogawa

Supplementary Figures and Table

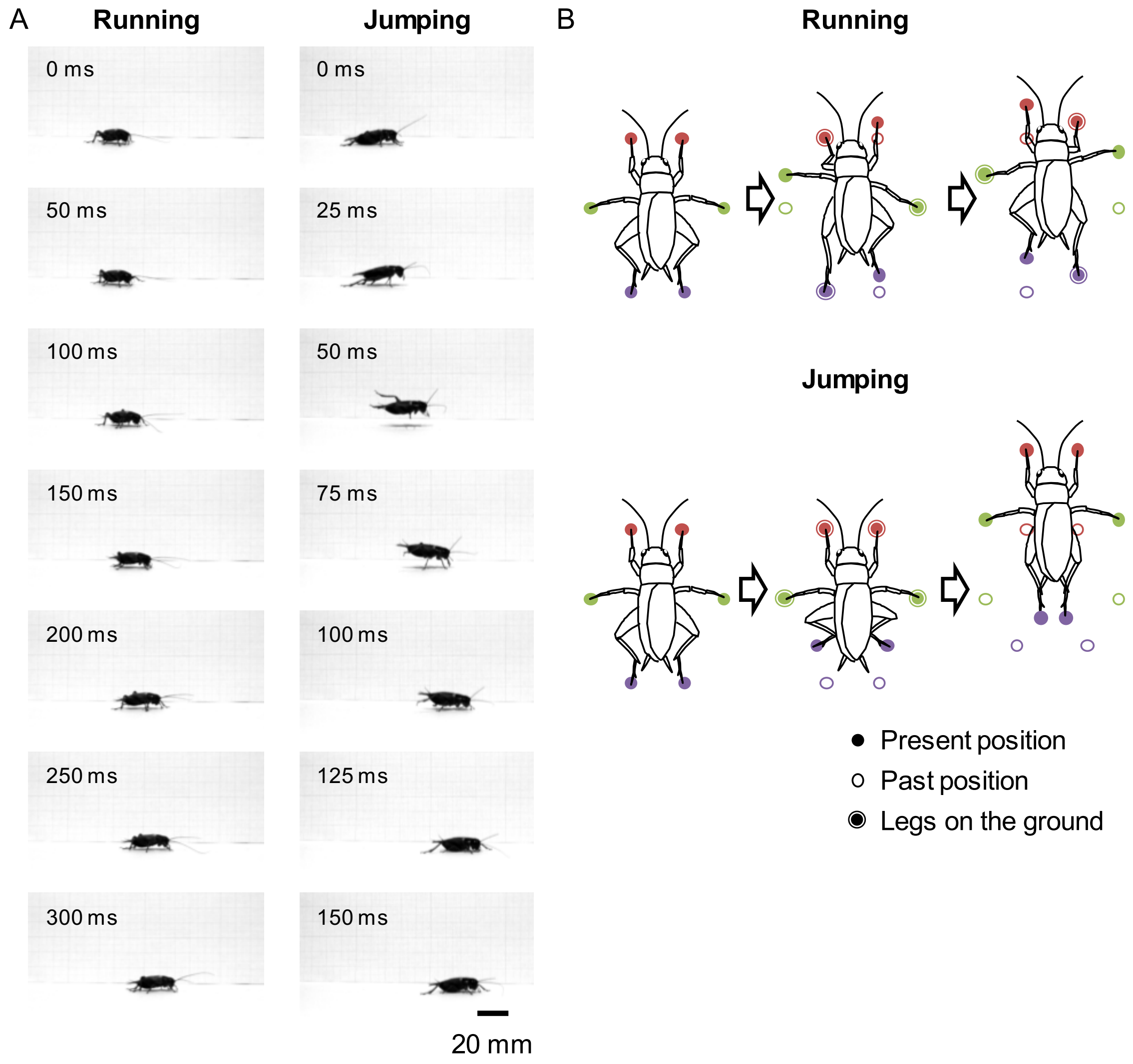

Supplementary Figure S1: Reactions in the wind-elicited escape behaviour of the cricket. (A) Typical running (left) and jumping (right) reactions elicited by air-current stimulation, which were horizontally recorded by a high-speed digital camera. An air current of 200-ms duration was applied to the cricket from the left side of the images. The numbers on the left of each image indicate the elapsed time after response onset. (B) Schematic diagrams of leg movements during running and jumping reactions, which were monitored vertically by a high-speed digital camera installed above the arena. During running, the left and right legs were moved alternately, and at least any three of legs contacted on the ground. For jumping, both hind legs were once flexed before kicking the

ground, and all six legs simultaneously left the ground. Filled circles indicate the current position of each tarsus and open circles indicate the previous position. So, double circles with inner filled and outer open circles indicates that tarsus position was not changed, meaning the leg did not move at that step.

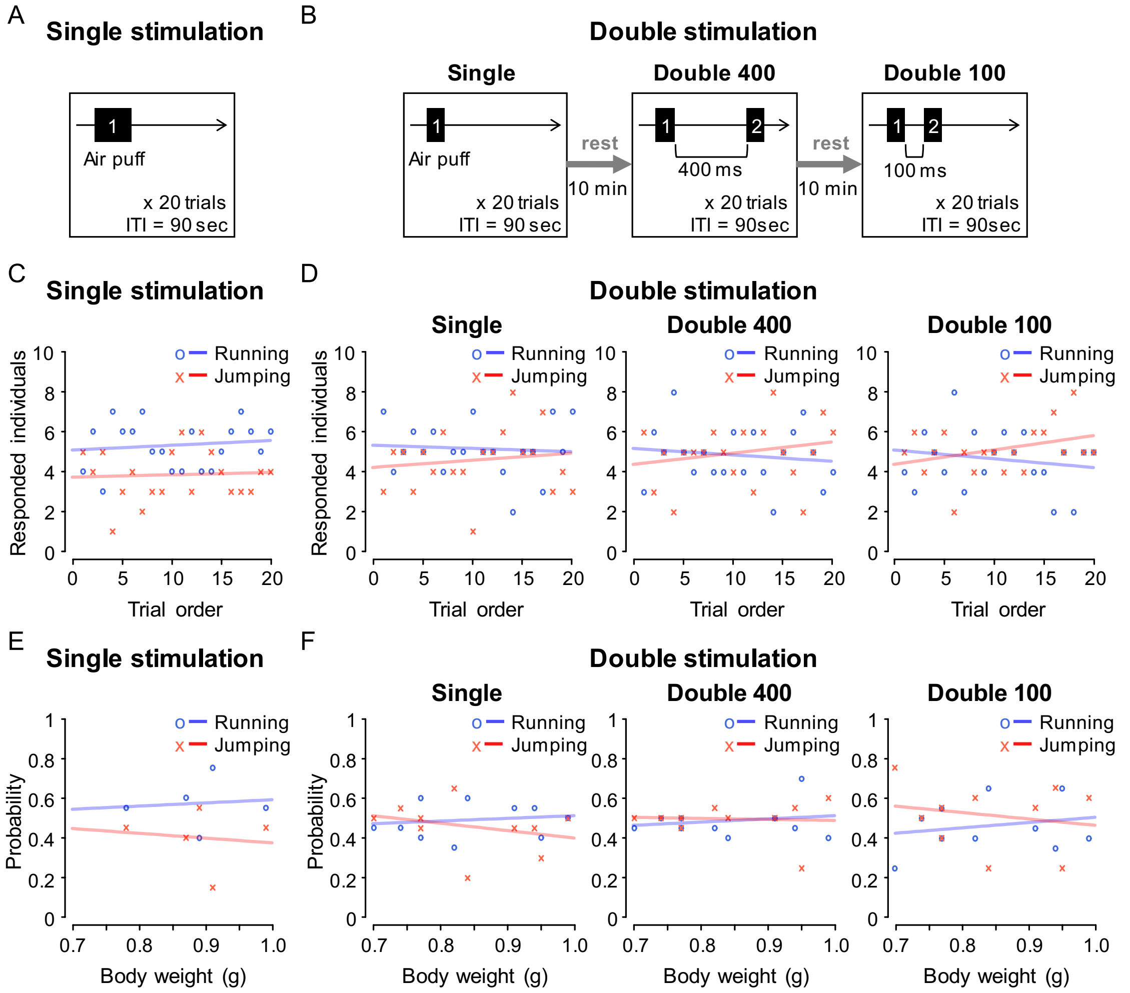

Supplementary Figure S2: Behavioural choice did not depend on trial order or weight of the animals. (A,B) Schematic diagrams of procedures in the single- (A) and double stimulus experiments (B). (C,D) Relationship between the trial order for each individual and the number of individuals responding to the first stimulus by running (blue "o") or jumping (red "x") in single- (C) and double stimulus experiments (D). Lines represent linear regression lines for the data for running (blue) or jumping (red). No significant relationship was indicated by linear regression analysis. $N_{animals}$ = 10 for each stimulation experiments. (E,F) Relationship between the weight of each individual and the probabilities of running (blue "o") and jumping (red "x") as the initial response in

single- (E) and double stimulus experiments (F). Lines represent linear regression lines for the data for running (blue) or jumping (red). No significant relationship was indicated by linear regression analysis. $N_{animals}$ = 5 and 10 for single- and double stimulus experiments, respectively.

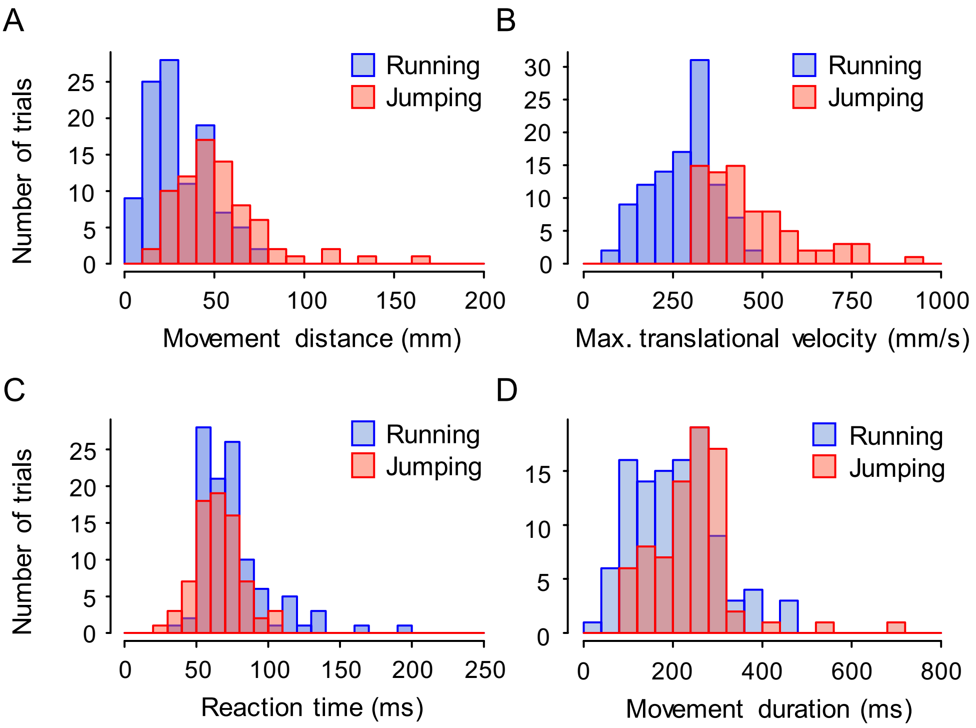

Supplementary Figure S3: Distribution of metric locomotor parameters. (A) movement distance, (B) maximum translational velocity, (C) reaction time, and (D) movement duration. Histograms indicates the number of trials in which the locomotion parameter values were measured for running (blue) and jumping (red).

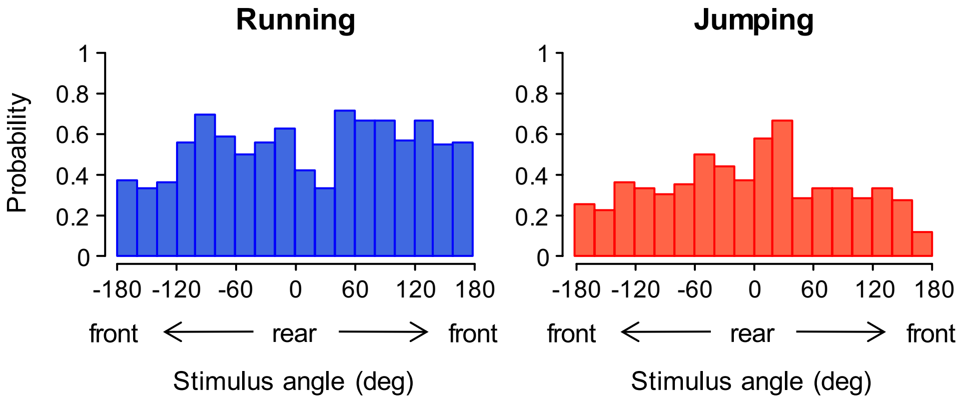

Supplementary Figure S4: Distribution of the angles of stimulus eliciting running and jumping. Histograms show the probabilities for running (blue) and jumping (red), which were calculated as ratio of the number of responding trials to all trials for every 30° of stimulus angles.

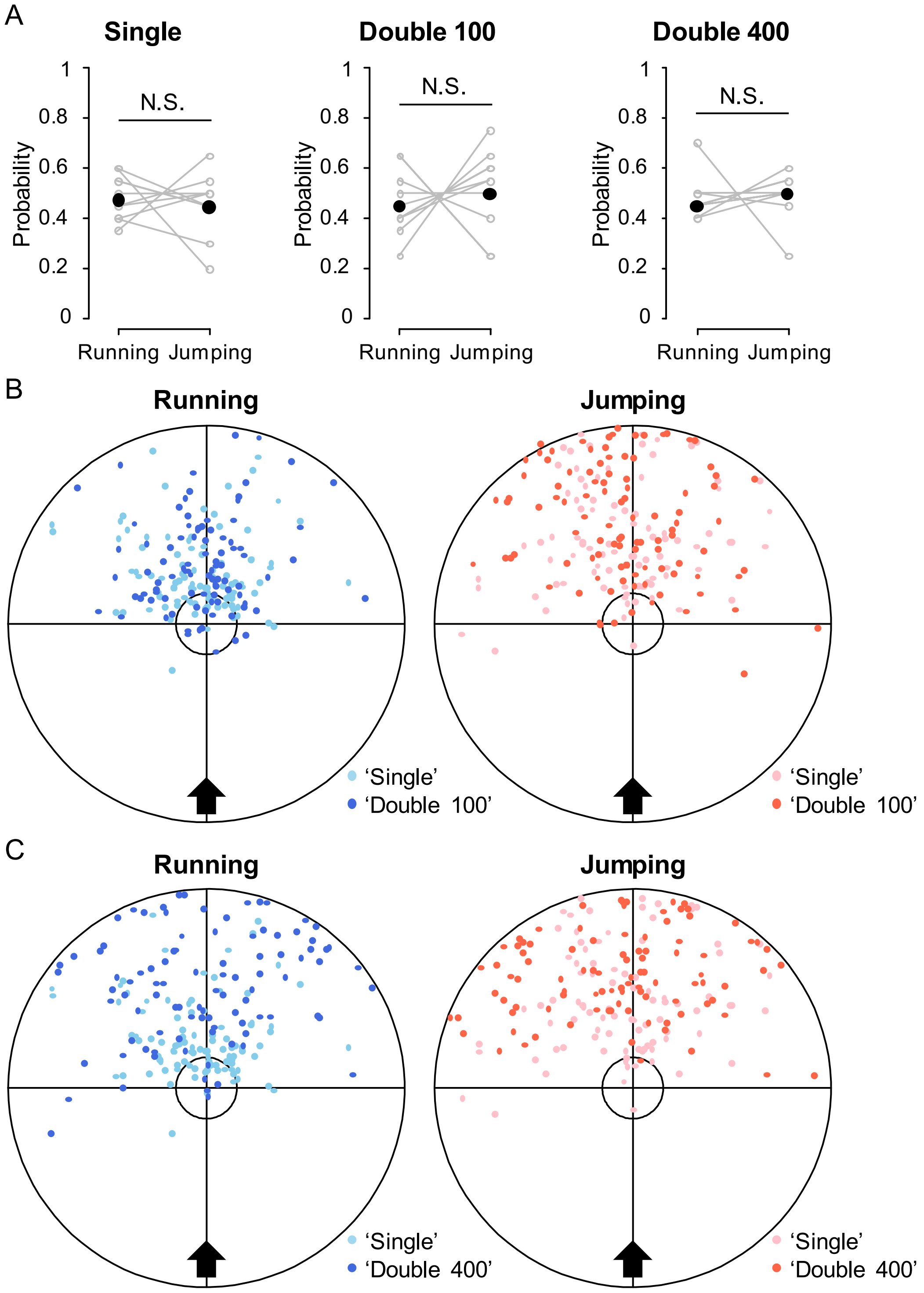

Supplementary Figure S5: Probabilities and distribution of the finish points in double stimulus experiment. (A) Probabilities of running and jumping as the first response in "Single" (left), "Double 100" (centre), and "Double 400" (right) stimulation

sessions. Gray open circles connected with lines represent the response probabilities for each individual, and black filled circles represent the mean of the probability for all individuals. N.S., not significant, Wilcoxon signed-rank test. N = 10 animals. (B,C) Spatial distributions of the finish points of whole escape locomotion in the trials. The data were divided by response to the first stimulus into running (left) and jumping (right). Thin coloured circles represent the trials in “Single”, and thick coloured circles represent the trials in “Double 100” (B) or “Double 400” (C). Black arrows indicate the direction of the air current stimuli. The centre small circle indicates starting locations of the crickets enclosed within a beaker, and the large circle indicates the wall of the experimental arena.

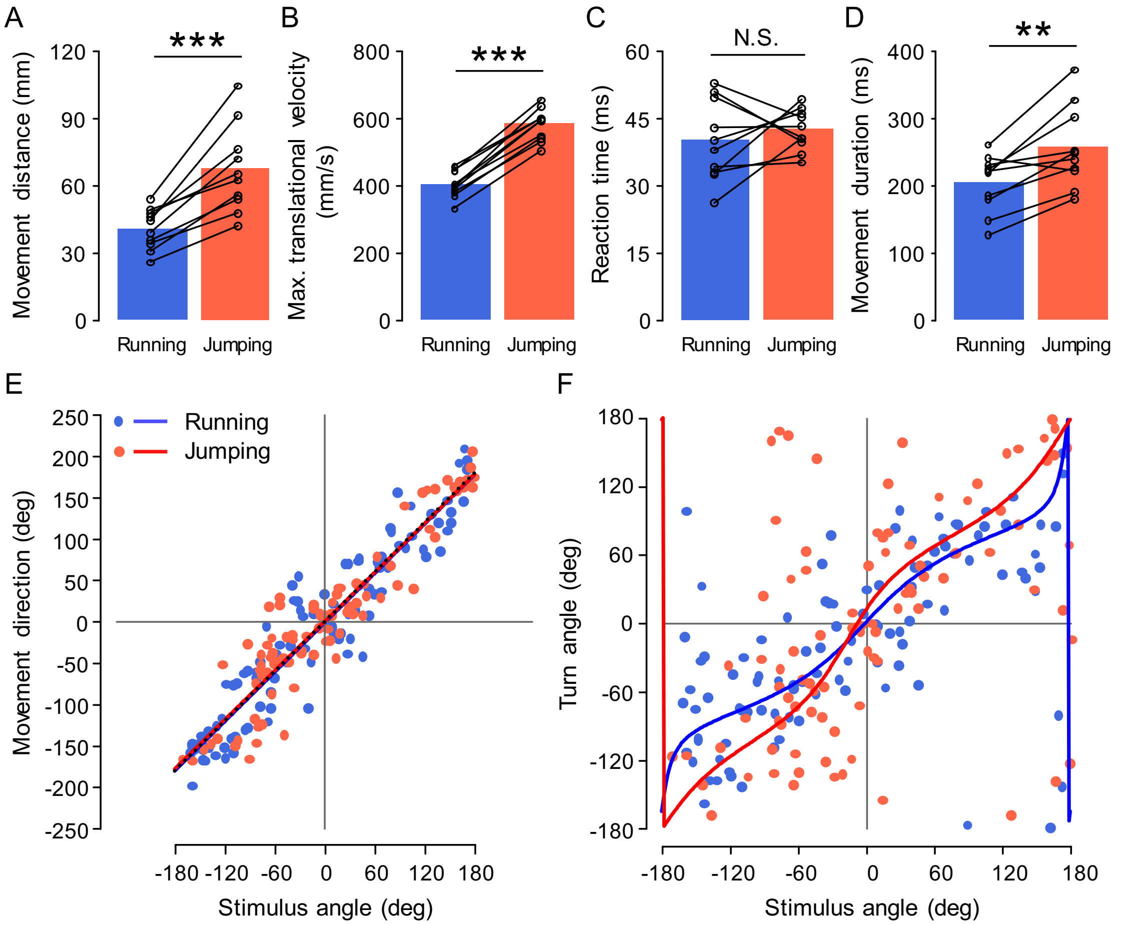

Supplementary Figure S6: Locomotive performances of running and jumping in "Single" session of double stimulus experiment. (A–D) Averages of mean movement distance (A), mean maximum translational velocity (B), mean reaction time (C), and mean movement duration (D) for all individuals. Black open circles connected with lines indicate the mean value for each individual. **p < 0.01, ***p < 0.001, N.S., not significant, paired t-test. N = 10 animals. (E) Relationships between the movement direction and stimulus angle in running (blue) and jumping (red). Lines represent linear regression lines for the data for running (blue) or jumping (red). Black dotted line indicates line of y = x. (F) Relationships between the turn angle and stimulus angle in running (blue) and jumping (red). Curves represent circular-circular regression curves. The differences in all motor parameters between running and jumping were consistent

with the results of single stimulus experiments in which the air-puff of 200-ms duration was used.

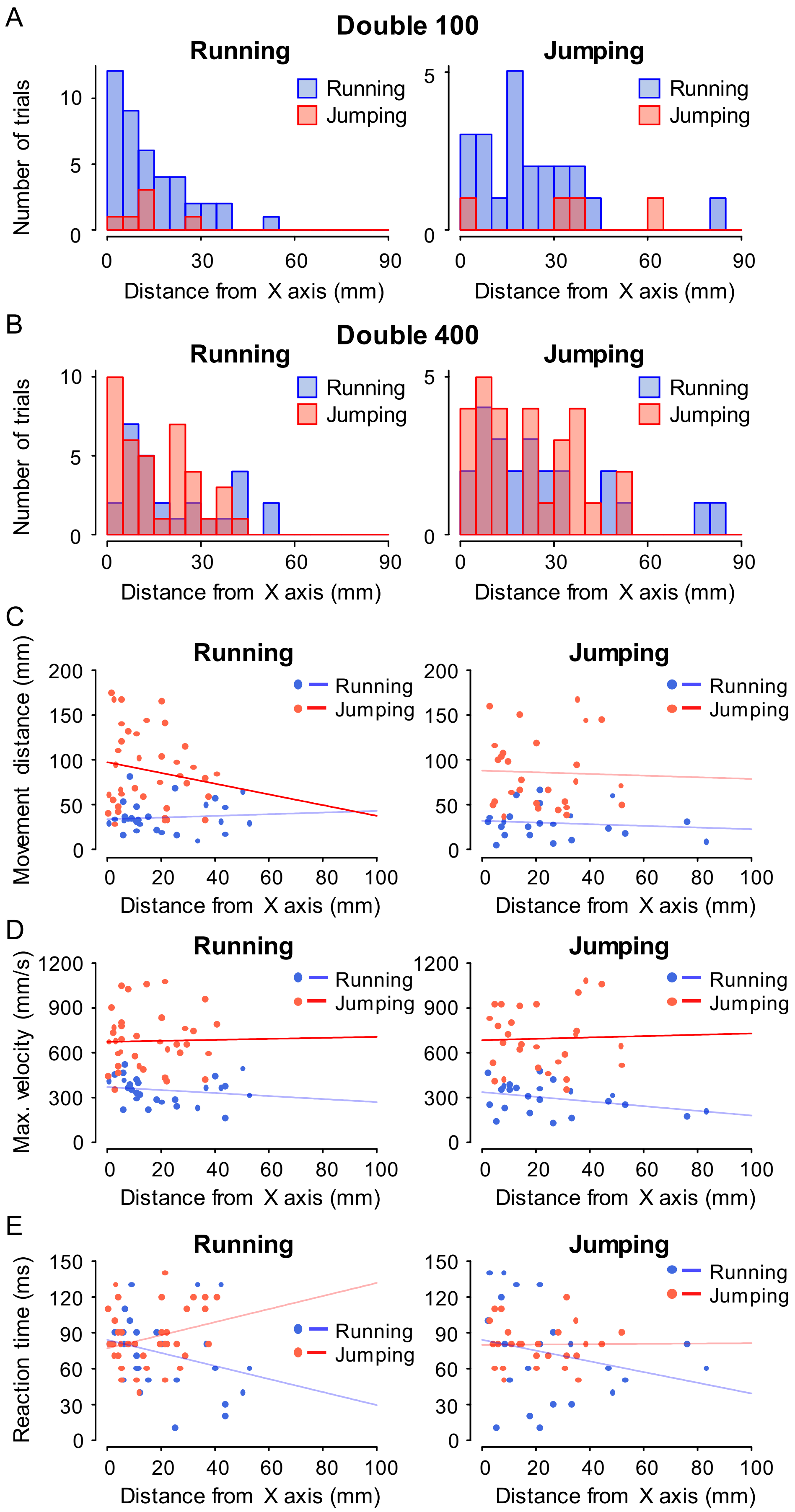
A
Double 100
Running
Jumping
Number of trials
Distance from X axis (mm)
B
Double 400
C
Movement distance (mm)
D
Max. velocity (mm/s)
E
Reaction time (ms)
Running
Jumping

Supplementary Figure S7: No effect of the position stimulated by the second puff in double stimulus experiments. (A,B) Distribution of the location where animals stimulated by the second stimulus. Histograms indicate the number of secondly-responding trials against the distance from the x-axis, which corresponds to airflow course, to the position stimulated by the second puff in “Double 100” (A) and “Double 400” (B). The data are divided by the first response into running (left) and jumping (right). (C–E) Relationship between the distance from the x-axis to the secondly-stimulated position and the metric locomotor parameters in the second response, including movement distance (C), maximum translational velocity (D), and reaction time (E) in “Double 400” session. The data are divided by the first response into running (left) and jumping (right). Lines represent linear regression lines for the data for running (blue) or jumping (red). Thick lines indicate statistical significance in linear regression analysis.

Supplementary Table S1. Numbers of responses for each experiment.

N_{trials} of responses for Fig. 1			
Stimulus	Running	Jumping	No response
Single stimulation	106	76	18
N_{trials} of responses for Figs. 2C and 3C			
Stimulus angle	Running	Jumping	No response
Behind	43	42	0
Lateral	38	20	3
Front	25	14	15
N_{trials} of first responses for Figs. 4 and 5			
Stimulus condition	Running	Jumping	No response
Single	102	91	7
Double 100	92	102	6
Double 400	95	98	7
N_{trials} of second responses after first running for Figs. 4 and 5			
Stimulus condition	Running	Jumping	No response
Double 100	42	6	44
Double 400	27	38	30
N_{trials} of second responses after first jumping for Figs. 4 and 5			
Stimulus condition	Running	Jumping	No response
Double 100	22	4	76
Double 400	23	28	47
